# Unraveling the genome-wide repertoire of the novel chromosomally encoded *mcr-8.6* gene variant in *Klebsiella michiganensis* isolated from manure

**DOI:** 10.3389/fmicb.2025.1673320

**Published:** 2025-12-03

**Authors:** Rani Rivière, Pedro Teixeira, Catarina Silva, Miguel Ramos, Elsa Dias, Vera Manageiro, Manuela Caniça

**Affiliations:** 1 National Reference Laboratory of Antibiotic Resistances and Healthcare Associated Infections, Department of Infectious Diseases, National Institute of Health Dr. Ricardo Jorge, Lisbon, Portugal; 2 Technology and Innovation Unit, Department of Human Genetics, National Institute of Health Dr. Ricardo Jorge, Lisbon, Portugal; 3 Laboratory of Biology and Ecotoxicology, Department of Environmental Health, National Institute of Health Dr. Ricardo Jorge, Lisbon, Portugal; 4 Centre for the Studies of Animal Science, Institute of Agrarian and Agri-Food Sciences and Technologies, University of Porto, Porto, Portugal; 5 Associate Laboratory for Animal and Veterinary Sciences (AL4AnimalS), Lisbon, Portugal; 6 Faculty of Veterinary Medicine, Center for Interdisciplinary Research in Animal Health (CIISA), University of Lisbon, Lisbon, Portugal

**Keywords:** *Klebsiella michiganensis*, *mcr-8.6*, manure, colistin resistance, mobile genetic elements, genomic island, chromosome-encoded resistance, Portugal

## Abstract

The increasing rates of colistin resistance worldwide poses a significant threat to public health. While the most commonly described variant is *mcr-1*, other variants such as *mcr-8* have been detected, typically associated with *Klebsiella pneumoniae*. However, little is known about the prevalence of *mcr-8* in other bacterial species and environmental reservoirs. This study aimed to characterize a novel *mcr-8* subvariant identified in a *Klebsiella michiganensis* strain isolated from manure in Portugal, collected during an annual longitudinal survey at an Open Air laboratory, as well as to depict its genomic context and potential mobility mechanisms. The strain was subjected to phenotypic susceptibility testing, whole-genome sequencing and hybrid genome assembly. *In silico* analysis included identification of resistance genes and mobile genetic element. The new gene variant *mcr-8.6* and its genetic environment were characterized. The F731 strain presented susceptibility to colistin with a MIC = 0.25 mg/L, despite carrying a novel *mcr-8* subvariant, *mcr-8.6*, which was located within a 61.6 kb chromosomal genomic island. This variant presented 23–24 amino acid substitutions compared to previous characterized MCR-8 proteins. The genomic island also harbored multiple insertion sequences (IS*110*, IS*66*, IS*3*), virulence factors, and metabolic and regulatory proteins, among others. Synteny analysis revealed high sequence identity between this genomic island and both chromosomal and plasmid regions from other bacterial strains isolated from different reservoirs worldwide, indicating prior mobility. Furthermore, other antimicrobial resistance genes were detected [e.g., *aph(3*′*)-la*, *bla*_*OXY–1–2*_], but no plasmid replicons were identified. This is the first report of a *mcr-8* gene in a *K. michiganensis*, as well as the first occurrence in Portugal. Although F731 remains colistin-susceptible, the presence of a novel *mcr-8.6* chromosomally encoded but located in a mobile genomic island underscores the risk of future horizontal gene transfer. These findings highlight the importance of further monitoring and continued surveillance in environmental and animal compartments in order to track the dissemination of antimicrobial resistance.

## 1 Introduction

Colistin, as a polymyxin, was moved by the World Health Organization (WHO) to the “Highest Priority Critically Important Antimicrobials for Human Medicine” since 2018 (World Health Organization, 2018). Its increasing use as one of the last-resort antimicrobials available for treating infections caused by carbapenem-resistant bacteria, simultaneously with its extensive use in veterinary medicine and animal production, have led to the emergence of colistin resistance (Bastidas-Caldes et al., 2022; Hatrongjit et al., 2024; Jian et al., 2024).

Colistin resistance, which is related to the modification of the negatively charged outer membrane lipopolysaccharides of Gram-negative bacteria, can be encoded through chromosomal mechanisms or plasmid-borne resistance genes (Snesrud et al., 2018; Zelendova et al., 2023). Particularly concerning are the plasmid-encoded *mcr* genes, as they allow the horizontal gene transfer (HGT) of colistin resistance among bacteria shared across humans, animals, and the environment (Liu et al., 2024).

The first plasmid-mediated colistin resistance gene, *mcr-1*, was detected in 2015 (Liu et al., 2016). Since then, twelve variants of this mobile gene have been detected in human (e.g., patients), animal (poultry, wild animals) and environmental samples from different sources, such as water matrices, agriculture, manure (Drali et al., 2018; Manageiro et al., 2020; Al-Mir et al., 2021; Rebelo et al., 2021; Torres et al., 2021; Teixeira et al., 2025). Indeed, the environment is considered an important reservoir of antimicrobial resistance genes (ARGs), and a potential amplifier of antibiotic resistance, where manure, as fertilizer, may contribute to the persistence of resistance genes, e.g., *mcr* genes, posing a potential public health risk (Lima et al., 2020; Teixeira et al., 2025). While *mcr-1* gene was first detected in *Enterobacterales* in China, within the six months following this description, plasmids carrying *mcr-1* gene were found in higher incidence in animal strains worldwide, including in the Europe (Mmatli et al., 2022).

The true prevalence of colistin resistance rates in *Enterobacterales*, however, remains unclear, because colistin susceptibility testing is not performed routinely in many settings and existing data are not fully representative (Prim et al., 2017). Some European countries might have implemented routine testing, particularly in reference laboratories or for multidrug-resistant isolates, however, there are yet methodological limitations and variability in surveillance practices. The last report (2014) from the European Antimicrobial Resistance Surveillance Network (EARS-Net), regarding colistin surveillance, collected antimicrobial resistance data from clinical invasive strains presented by European countries; the data showed that countries with high percentages of carbapenem resistance also reported elevated numbers of clinical strains resistant to polymyxin, indicating a further loss of effective antimicrobial treatment options. Specifically, among all carbapenem-resistant *K. pneumoniae* and *Escherichia coli* clinical strains, 29.0% and 4.4% were co-resistant to polymyxin, respectively (European Centre for Disease Prevention and Control, 2015).

In Portugal, colistin resistance among clinical *Enterobacterales* isolates remains relatively low compared to some Southern European countries, as Greece and Italy, where 26.0% (2014) and 46% (2018) of *K. pneumoniae* were, respectively, resistant to polymyxin (Shahzad et al., 2023). The frequency of clinical invasive colistin-resistant *E. coli* strains, in Portugal, remained low and sporadic over the years 2015 and 2019, however, slightly decreasing in 2024; for clinical invasive *K. pneumoniae* strains, colistin resistance showed greater variability (personal communication, M. Caniça). Until date, *mcr-1* is the most frequently detected variant in Portugal in different reservoirs (Mendes et al., 2018; Manageiro et al., 2020; Portes et al., 2022; Amaro et al., 2023), *mcr-9* in patients and fish farming (Manageiro et al., 2022; Silveira and Pista, 2023), and *mcr-4* have also been detected but restricted to pigs (Lima et al., 2022; Amaro et al., 2023).

The *mcr-8* gene, like other *mcr* variants, alters the bacterial outer membrane, and its origin has been linked to an environmental bacteria, *Kosakonia sacchari*, which carries a putative chromosomal gene encoding a protein that shares 70% amino acid identity with MCR-8 (Fournier et al., 2021). The *mcr-8* variant has been identified in several *Enterobacterales* species, mostly in *K. pneumoniae*, *Klebsiella quasipneumoniae*, *Raoultella ornithinolytica*, in every continent, although the majority of reports originate from Asia (Wang et al., 2018, 2019; Hadjadj et al., 2019; Mo et al., 2024; Zhang et al., 2025). However, *mcr-8* has not been detected in *Klebsiella michiganensis*, which belongs to the *Klebsiella oxytoca* complex (Shibu et al., 2021; Prah et al., 2022). This is alarming, as these bacteria are reported in clinical settings, carrying clinically relevant ARGs, and are responsible for bloodstream infections, nosocomial infections and severe septicaemia, especially in premature infants and immunocompromised patients (Hrenovic et al., 2016; Zheng et al., 2018; Seiffert et al., 2019; Campos-Madueno et al., 2021; Li et al., 2022; Prah et al., 2022; Simoni et al., 2023; Zhang et al., 2023; Xu et al., 2024). One of the most concerning threats arises when plasmid-mediated *mcr* genes co-occur with other ARGs, whether plasmid-borne or chromosomal encoded, within the same bacterial pathogen (Long et al., 2019; Zhou et al., 2022).

The aim of this study was to evaluate the potential evolutionary relationship and genome-wide repertoire of a novel subvariant of the *mcr-8* gene detected in *K. michiganensis*, isolated from manure, particularly in comparison to previously reported *mcr-8* subvariants and their association with mobile genetic elements (MGEs) potentially involved in the acquisition of *mcr-8.6*.

## 2 Materials and methods

### 2.1 Sampling, isolation and bacteria identification

The strain F731 was isolated from manure, composed by animal feces, mostly of pigs, collected during an annual longitudinal study in December 2020 at an experimental agricultural and agri-food production station in Portugal (Open Air Laboratory), located in Santarém, Vale de Santarém, 75 km from Lisbon. Sample collection, transport and storage procedures were previously described.^1^,^2^ Then, the manure was mixed with buffered peptone water and inoculated on MacConkey agar containing 0.5 mg/L of colistin, followed by isolation on simple agar medium overnight at 35 °C.^3^ Species identification was performed using VITEK^®^2 Automated Identification System (BioMérieux, Marcy-l’Étoile, France) and MALDI-TOF (BioMérieux, Marcy-l’Étoile, France). The strain was preserved at −80 °C in Trypticase soy broth with 20 % glycerol.

### 2.2 Antimicrobial susceptibility testing

Antimicrobial susceptibility testing was performed using the disk diffusion method. A total of 13 antimicrobial were tested, covering the following classes: β-lactams, including amoxicillin/clavulanic acid (20+10 μg), cefotaxime (30 μg), ceftazidime (30 μg), cefepime (30 μg), piperacillin/tazobactam (36 μg), aztreonam (30 μg), ertapenem (10 μg), imipenem (10 μg), meropenem (10 μg), and cefoxitin (30 μg); aminoglycosides represented by gentamicin (10 μg); fluoroquinolones represented by ciprofloxacin (5 μg); and sulfonamides, represented by co-trimoxazol (25 μg). Resistance mechanisms were inferred through synergy testing using the following compounds: boronic acid to detect class A carbapenemases; cloxacillin alone to detect AmpC β-lactamases; cloxacillin in combination with amoxicillin/clavulanic to identify AmpC β-lactamases and ESBLs; dipicolinic acid with meropenem to detect metallo-β-lactamases; faropenem to indicate carbapenemase production; and temocillin to infer the presence of OXA-48-producing isolates (disks from Bio-Rad, Marnes-la-Coquette, France). *E. coli* ATCC 25922 was used as control strain and the results were interpreted according to the critical diameters defined by EUCAST v.2025 (European Committee on Antimicrobial Susceptibility Testing; EUCAST, 2025).

The minimum inhibitory concentration (MIC) for colistin was determined by the microdilution method using an in-house broth used in a 96-well microdilution plate prepared at the National Institute of Health Dr. Ricardo Jorge (INSA). EUCAST guidelines and EN/ISO 17025 standards were followed. *E. coli* ATCC 25922 and *E. coli* NCTC 13846 were used as control strains for susceptibility and resistance, respectively, to colistin. The results were interpreted based on clinical breakpoints defined by EUCAST guidelines (2025) for *Enterobacterales* (susceptible ≤ 2 mg/L; resistant > 2 mg/L).^4^

### 2.3 Whole-genome sequencing

Genomic DNA was extracted from a freshly grown overnight culture using the MagNA Pure 96 instrument (Roche, Manheim, Germany) and quantified with a Qubit™ 4 fluorometer (Thermo Scientific, Waltham, USA) using the Qubit dsDNA BR Assay Kit, following the manufacturer’s instructions.

#### 2.3.1 Illumina short-read sequencing

The sequencing library was prepared using the Nextera XT Library Preparation Kit (Illumina, San Diego, USA) and sequenced on an Illumina MiSeq platform with 150 bp paired-end reads, following the manufacturer’s guidelines. Sequencing data was processed for quality control of raw reads, *de novo* assembly, and species confirmation using INNUca (v4.2.2).^5^ The INNUca pipeline encompasses read quality assessment using FastQC (v0.11.5),^6^ trimming with Trimmomatic (v0.38) (Bolger et al., 2014), and genome assembly with SPAdes (v3.14.0) (Bankevich et al., 2012). Completeness of the genome was evaluated using BUSCO (v5.5.0) (Simão et al., 2015).

#### 2.3.2 Nanopore long-read sequencing

The MinION library was prepared using the SQK-RBK114.24 Rapid Barcoding Kit and a MinION R10.4.1 flow cell, and then sequenced on a Mk1C device (Oxford Nanopore Technologies, Oxford, UK) for 72 h. Basecalling and barcode trimming were performed on the sequencing device, following the model Dorado (v7.6.7). Overall read quality was assessed using pycoQC (v2.5.2) (Leger and Leonardi, 2019) and Nanoplot (v1.42.0) (De Coster and Rademakers, 2023).

### 2.4 *In silico* analysis of genomic data

Hybrid *de novo* assembly was performed using Hybracter (v0.7.3) pipeline (Bouras et al., 2024), followed by analyses of the hybrid assembled genome. Species confirmation was performed based on Average Nucleotide identity (ANI) using FastANI (v1.33) (Jain et al., 2018) against *Klebsiella* spp. reference genomes from NCBI.^7^ Detection of ARGs, plasmids replicons and virulence factors were performed using abriTAMR (v1.0.14) (Sherry et al., 2023) and ABRicate (v1.0.1).^8^ ABRicate incorporates the following databases: NCBI, ResFinder, CARD, PlasmidFinder, VFDB (17.09.2024). Genomic islands were searched using the tool IslandViewer4 (Bertelli et al., 2017). The genome sequence was annotated using Prokaryotic Genome Annotation Pipeline (PGAP-2023-10-03.build7061) (Tatusova et al., 2016).

For genome comparisons, BLASTn was used (Altschul et al., 1990). The genetic context of *mcr* genes was mapped and analyzed using pyGenomeViz (v0.4.4),^9^ and the final figure was edited for layout and labeling in Inkscape (v1.4.2).

The amino acid sequences of MCR variants were downloaded from NCBI^10^ and compared with the variant detected in this study using Jalview (v2.11.4.1) (Waterhouse et al., 2009). A Neighbor Joining tree (BLOSUM62) was generated and exported in Newick format and visualized on iTOL (v7) (Letunic and Bork, 2021).

### 2.5 Nucleotide sequence accession numbers

The new *mcr-8.6* nucleotide sequence was submitted to NCBI under the accession number PV035885 and the total genome sequence of F731 was deposited on GenBank under the accession number CP182858.

## 3 Results

### 3.1 Species identification and phenotypic characterization

F731 was initially identified as *K. oxytoca* by the VITEK^®^2 Automated Identification System and MALDI-TOF. However, ANI analysis, using the single contig from the hybrid genome assembly with 92× coverage, a total length of 6,048,658 bp, and a GC content of 55.85%, confirmed the strain as *K. michiganensis* with 99.2% identity compared to reference *Klebsiella* genomes.

Regarding the antimicrobial susceptibility testing, the strain presented resistance to the antimicrobial class aminoglycoside (gentamicin), and susceptibility to all other classes tested, including β-lactams (amoxicillin/clavulanic acid, cefotaxime, ceftazidime, aztreonam, cefepime, piperacillin/tazobactam, ertapenem, meropenem, imipenem, and cefoxitin), fluoroquinolones (ciprofloxacin) and sulfonamides (co-trimoxazol). No synergy was observed between antimicrobials and the respective inhibitors referred in material and methods section (boronic acid, cloxacillin and dipicolinic acid). The MIC for colistin was 0.25 mg/L, classifying the strain as susceptible according to EUCAST breakpoints.

### 3.2 ARGs, virulence factors and plasmids

Strain F731 harbored multiple ARGs and virulence factors (Supplementary Tables 1, 2). The ARGs and their variants identified included *bla*_OXY–1–2_ (encoding a class A β-lactamase capable of hydrolysing penicillin and 1st generation cephalosporins); *aph(3*′*)-la* (encoding for an aminoglycoside phosphotransferase); *oqxA10* and *oqxB9* (components of an efflux pump that mostly leads to diminished susceptibility to quinolones and chloramphenicol); and *fosA9*-type that confers resistance to fosfomycin. A novel subvariant of *mcr-8*, the *mcr-8.6*, was also identified, as well as *eptB* and *arnT* that are all involved with the reduction of colistin efficacy and binding. In addition, genes were detected that confer resistance to multiple classes as they are efflux pumps or regulators that influence other ARGs, such as *acrA*, *acrB*, *acrD*, *mdtB*, *mdtC*, *mdtQ*, *emrD*, *emrR*, *marA*, *ramA*, *cpxA*, *H-NS* and *CRP*, as well as genes that lead to modifications or losses in outer membrane components which alter bacteria permeability, including *ompK37*, *ompA*, *lptD*, *kpnE*, *kpnF*, *kpnG*, *kpnH* and *msbA*.

Genes conferring metal resistance were also detected, including *arsR* and *arsC* that confer resistance to arsenic, *terD*, *terC* and *terB* to tellurite, leading to the capability of surviving in toxic metal concentrations. Notably, the strain also harbored virulence factors, such as *ybtA*, *ybtE*, *ybtP*, *ybtQ*, *ybtS*, *ybtT*, *ybtU*, *ybtX*, *irp1*, *irp2* involved in siderophore-dependent iron transport system, as well as *fyuA*, *entA* and *entB* for iron acquisition (Geoffroy et al., 2000; Penwell et al., 2012); *mgtB* related to Mg^2 +^ transport, *yagZ/ecpA* involved with adhesion, and *ompA* which is also considered a virulence factor that can also lead to adherence, invasion and biofilm formation (Scheller et al., 2021). No plasmids were detected.

### 3.3 A new variant of the *mcr-8* gene

**FIGURE 1:**
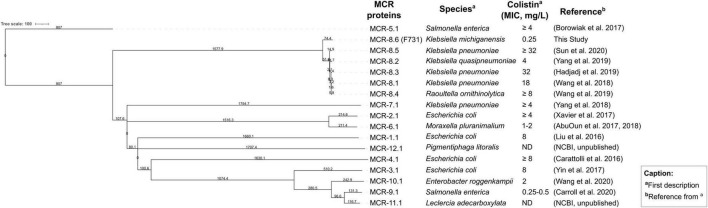
Maximum-likelihood phylogenetic tree based on amino acid sequences, comparing the F731 MCR-8 with known other MCR-8 variants [MCR-8.1 (WP_114699275), MCR-8.2 (WP_072310976), MCR-8.3 (WP_150823497), MCR-8.4 (WP_118860654), MCR-8.5 (QKV49902)] and a representative from each MCR variant [MCR-1.1 (WP_049589868), MCR-2.1 (WP_065419574), MCR-3.1 (WP_039026394), MCR-4.1 (WP_099156046), MCR-5.1 (WP_053821788), MCR-6.1 (WP_099982813), MCR-7.1 (WP_104009851), MCR-9.1 (AYW01299), MCR-10.1 (WP_023332837), MCR-11.1 (QEY54480)], MCR-12.1 (NG_245195). The tree was constructed using a maximum-likelihood method with branch length (path of transmission of genetic information from one generation to the next) values indicated at each node. Only one representative subvariant per MCR-8 family was included to keep the tree readable, using the RefSeq protein sequence from NCBI as the reference. The table to the right summarizes the host species, minimum inhibitory concentrations (MICs) for colistin, and references for each MCR variant (Xavier et al., 2016; Liu et al., 2016; AbuOun et al., 2017; Borowiak et al., 2017; Yin et al., 2017; Carattoli et al., 2017; Yang et al., 2018, 2019; Wang et al., 2018, 2019, 2020; Carroll et al., 2019; Hadjadj et al., 2019; Sun et al., 2020).

Analysis with both abriTAMR and ABRicate identified the *mcr-8.1* gene with values of 99.94% coverage and 95.41% identity, suggesting the presence of sequence divergence compared to the reference, likely due to mutations or other genetic variations. To further assess these differences, a maximum likelihood phylogenetic tree was constructed, that included F731 *mcr-8*-type along with all known *mcr-8* subvariants (*mcr-8.1*, *mcr-8.2*, *mcr-8.3*, *mcr-8.4*, *mcr-8.5*) and one representative of all other *mcr* gene families (*mcr-1.1*, *mcr-2.1*, *mcr-3.1*, *mcr-4.1*, *mcr-5.1*, *mcr-6.1*, *mcr-7.1*, *mcr-9.1*, *mcr-10.1*, *mcr-11*, *mcr-12*) (Figure 1). F731 *mcr* gene consistently clustered within the *mcr-8* clade, indicating this may represent a novel subvariant. This conclusion is reinforced by the branch length value of 75.2, equalling 0.752 substitutions per alignment position, separating F731 *mcr-8*-type from other *mcr-8* subvariants, which indicates a clear genetic distance within this group.

Further evidence supporting the novelty of this variant was provided by the differences observed at the protein level through amino acid sequence alignment of MCR-8 subvariants (Table 1; Supplementary Table 3). The MCR-8 protein identified in F731 differed from previously described variants by 23–24 amino acids (Table 1). Additionally, it had a two amino acid insertion (Ser-566 and Lys-567), which is only present in the subvariant from CTHL.F3a and has not yet been characterized. Overall, these amino acid differences collectively allowed to identify a novel variant of the MCR-8 protein, which encodes to a new *mcr-8*-type gene, which was submitted to NCBI and classified as *mcr-8.6*.

**TABLE 1:** Amino acid variations of the MCR-8.6 subvariant compared with other previous described MCR-8 subvariants.

	Amino acid residues at positions where F731 differs from other MCR-8 variants																																					
Subvariant	39	41	51	134	141	146	149	169	216	219	232	296	301	348	356	365	391	399	404	421	444	466	480	488	500	504	508	512	513	520	535	549	553	555	564	565	566	567
MCR-8.1	V	V	A	V	L	N	G	A	I	S	A	N	V	I	G	N	T	T	S	V	R	S	N	F	K	E	G	K	Q	Y	K	H	S	A	N	G	–	–
MCR-8.2	V	V	V	V	L	N	G	A	I	S	S	N	V	I	G	Y	T	T	S	V	R	S	K	F	K	E	G	K	Q	Y	K	H	S	A	N	G	–	–
MCR-8.3	V	V	A	V	L	N	G	A	I	S	A	N	V	I	G	N	K	T	S	V	R	S	N	F	K	E	G	K	Q	Y	K	H	S	A	N	G	–	–
MCR-8.4	V	V	A	V	L	N	G	A	I	S	A	N	V	I	G	N	T	T	R	V	R	S	N	F	K	E	G	K	Q	Y	K	H	S	A	N	G	–	–
MCR-8.5	V	V	V	V	L	N	G	A	I	S	S	N	V	I	G	N	T	T	S	V	R	S	N	I	K	E	G	K	Q	F	K	H	S	A	D	G	–	–
MCR-8-type CTHL.F3a	V	V	V	M	L	I	R	T	T	R	A	S	I	V	G	H	T	A	S	I	H	R	N	F	R	V	S	N	Q	Y	K	R	T	A	M	V	S	K
MCR-8.6 (F731)	A	F	V	V	S	I	R	T	T	R	A	S	V	V	S	H	T	T	S	V	R	R	N	F	R	A	S	N	K	Y	N	H	S	S	M	V	S	K

In red: amino acid substitutions; In blue: phosphoethanolamine transferase N-terminal domain; In green: sulfatase N-terminal domain.

### 3.4 Identification of a putative genomic island carrying *mcr*

**FIGURE 2:**
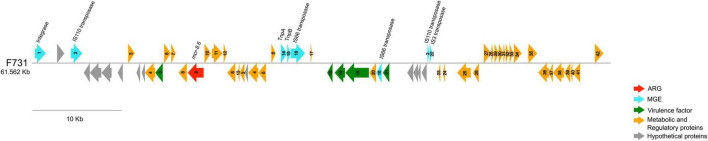
Genomic island carrying the *mcr-8.6* gene. Each arrow represents a predicted open reading frame and its direction of transcription and in gray, the hypothetical proteins. The genes integrated in the genomic island are: 1- integrase, 2- IS110 transposase, 3- DUF2975 domain-containing protein, 4- serine hydrolase domain-containing protein, 5- MipA/OmpV family protein, 6- SDR family oxidoreductase, 7- GyrI-like domain-containing protein, 8- ATP-grasp fold amidoligase family protein, 9- *mcr-8.6*, 10- transcriptional factor, 11- HAMP domain containing sensor histidine kinase, 12- diacylglycerol kinase, 13- helix-turn-helix transcriptional regulator, 14- IS66-like element accessory protein TnpA, 15- IS66-like element acessory protein TnpB, 16- IS66 transposase, 17- carbohydrate-binding module, 18- fimbrial protein, 19- fimbrial pilus, 20- EAL domain-containing protein, 21- EcsC family protein, 22- IS3 transposase, 23- AlpA family transcriptional regulator, 24- AlpA family phage regulatory protein, 25- YfjI family protein, 26- inovirus Gp2 family protein, 27- DUF932 domain-containing protein, 28- DUF905 domain-containing protein, 29- antirestriction protein, 30- DNA repair protein RadC, 31- DUF987 domain-containing protein, 32- type IV toxin-antitoxin system YeeU antitoxin, 33- TA system toxin CbtA family protein, 34- DUF4942 domain-containing protein, 35- aldo/keto reductase, 36- acetyl-CoA C-acetyltransferase, 37- carboxy mycolactone decarboxylase, 38- MFS transporter 39- HAD-IB family hydrolase, 40- tautomerase family protein, 41- AfsA-related hotdog domain-containing protein, 42- LysR family transcriptional regulator.

The *mcr-8.6* is located within a genomic island with a length of 61.562 Kb (Figure 2). This region contains multiple MGEs, including an integrase and an IS*110* transposase flanking the *mcr* gene. Further along the genomic island, other MGEs are present, such as IS*66*, IS*3*, and another IS*110*. Adjacent to *mcr-8.6*, several other genes with diverse functions were detected, including ATP-binding cassette transports, transcriptional regulators, serine hydrolase domain-containing proteins, and potential virulence factors, such as fimbrial pilus enabling adhesion to surfaces and host cells.

**FIGURE 3:**
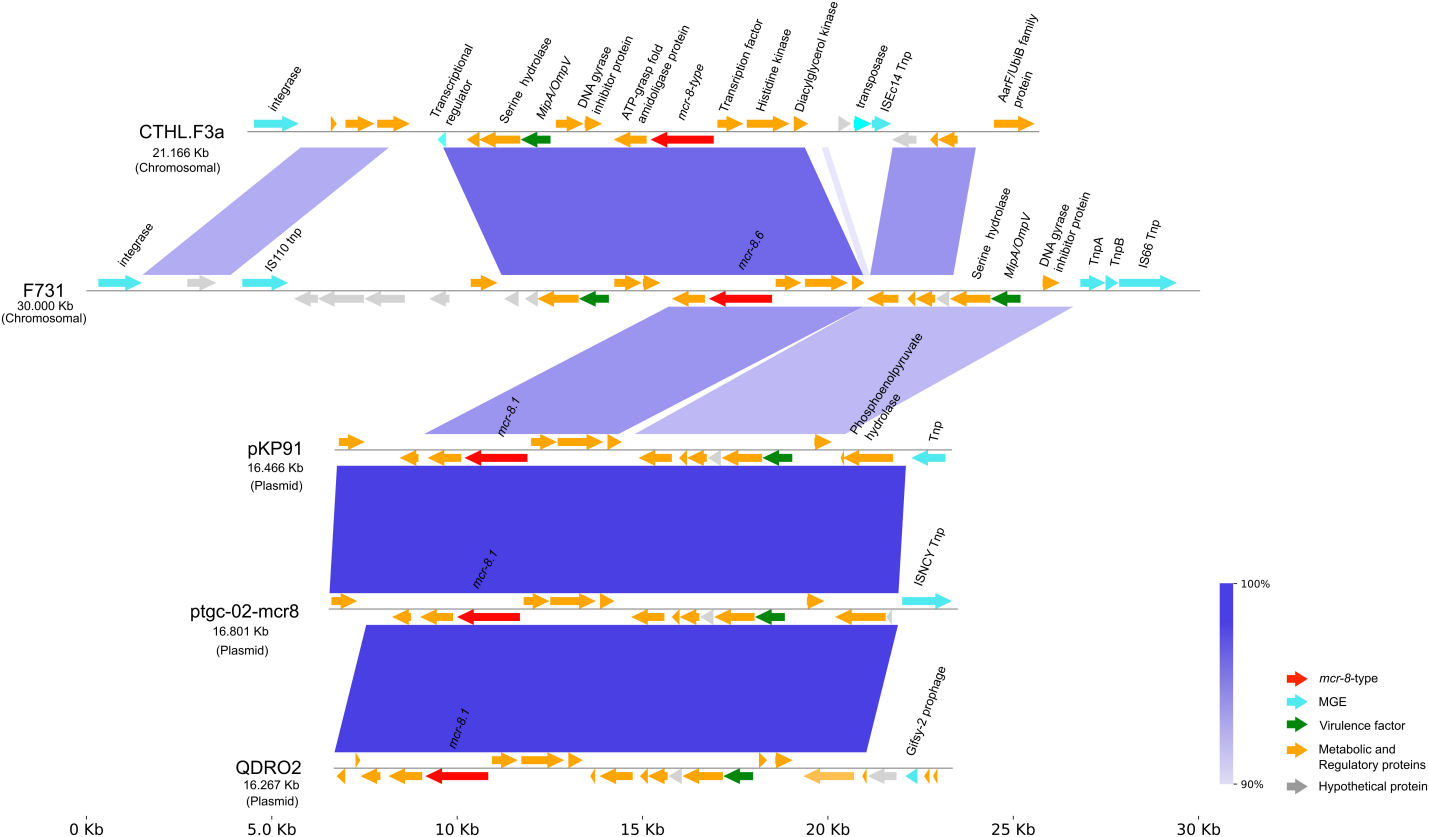
Genetic context of the F731 region containing the *mcr-8.6* gene compared against chromosomal [CTHL.F3a (CP082360)] and plasmids sequences [pKP91 (NZ_MG736312), tgc-02-mcr8 (NZ_CP132218), and QDRO2 (NZ_QWIW00000000)]. Color coded arrows represent the functional category of each gene, and the shaded regions highlight the presence of areas with high identity.

To further characterize the genomic island, synteny analyses were performed (Figure 3). Comparison of F731 genomic island with both chromosomal (e.g., CTHL.F3a) and plasmid sequences (e.g., pKP91, ptgc-02, QDRO2) revealed homologous regions with high sequence identity (90%–100%). The chromosomal sequences of *Klebsiella* sp. CTHL.F3a, isolated from a cabbage in Hong Kong, displayed a high degree of homology with the genes flanking the *mcr-8.6* gene in the F731 strain. However, CTHL.F3a harbors a different MCR-8-type subvariant, which differs by 16 amino acids.

Similarly, the region adjacent to *mcr-8.1* subvariant (length between 16,267 and 16,801 base pairs), and present in plasmids pK91, QDRO2 and ptgc-02-mcr, is highly conserved and also found adjacent to *mcr-8.6* in F731. The slightly differences observed correspond mainly to intergenic regions. This conserved region was also identified in 96 other plasmids/chromosome, according to BLASTN results (Supplementary Table 4), as well as in the chromosome of strain CTHL.F3a (Figure 3). The similarity between the genomic island in F731 and the regions referred above suggests that this island shares a common genetic backbone with other bacterial strains.

## 4 Discussion

In this study, we identified a novel *mcr-8* subvariant (*mcr-8.6*) located within the chromosome of a *K. michiganensis* strain, recovered from manure collected in an Open Air Laboratory in Portugal. To the best of our knowledge, this is the first report of an *mcr-8*-positive strain in Portugal and the first identification of this gene in a *K. michiganensis* worldwide. Our results demonstrate that the *mcr-8.6* is located within a genomic island that shares a common genetic backbone with other *mcr-8*-type gene harboring MGE. Additionally, the MCR-8 protein exhibits significant amino acids variations in regard to previously detected MCR-8 subvariants.

*K. michiganensis*, where *mcr-8.6* was identified, belongs to the *K. oxytoca* complex, and is often misidentified, which occurred in this study, as *K. michiganensis* is rarely included in MALDI-TOF databases (Shibu et al., 2021; Prah et al., 2022). This is alarming, as this bacterium is an emerging nosocomial pathogen that has been reported also in clinical settings, carrying clinically relevant ARGs, such as *bla*_KPC–3_, *bla*_VIM–1_, *bla*_SIM–1_, *mcr-9* (Hrenovic et al., 2016; Zheng et al., 2018; Seiffert et al., 2019; Campos-Madueno et al., 2021; Li et al., 2022; Prah et al., 2022; Simoni et al., 2023; Zhang et al., 2023). Although F731 carried numerous genes that are involved with antimicrobial resistance, only a few were phenotypically relevant, resulting in a matching susceptibility profile. Among them, *aph(3*′*)-la* suggests to be responsible for the phenotypic resistance to aminoglycoside. Meanwhile, the susceptible phenotypic corresponding to the *bla*_OXY–1–2_ gene was expected, as it is a chromosomally encoded β-lactamase gene that is constitutively expressed at low levels in the *Klebsiella* genus, which is insufficient to hydrolyse the antimicrobial effectively unless in the presence of mutations in the promotor region (Yang et al., 2022).

By contrast, the colistin MIC susceptible result was less expected (MIC = 0.25 mg/L), as the other *mcr-8* gene subvariants generally confer a resistant phenotype with MICs ≥ 4

(Wang et al., 2018; Ge et al., 2022; Cahill et al., 2023; Figure 1). Discrepancies between the detection of some variants of *mcr* genes and a susceptible phenotype to colistin have been previously reported, depending on bacterial species, serotype, and/or source of isolation (Bertelloni et al., 2022; Gaballa et al., 2023). To the best of our knowledge, previously described *mcr-8*-positive strains have consistently exhibited a colistin resistance phenotype. However, most *mcr-8* genes detected so far are plasmid-borne and might exist in multiple copies per cell, which can enhance resistance phenotype (Wang et al., 2018, 2019; Hadjadj et al., 2019; Salloum et al., 2020; Liu et al., 2022), unlike the chromosomally encoded gene in F731. In fact, Zhang et al. (2017), observed that the chromosomally encoded *mcr-1* had lower expression levels than most plasmid-mediated *mcr-1* levels (Zhang et al., 2017). The integration into the chromosome can lead to the downregulation of both gene expression levels and/or regulatory elements, as well as due to the broader context background of the bacterial strain that can also influence whether a resistance gene leads to an observable resistant phenotype (Suzuki et al., 2014; Zhang et al., 2017; Gaballa et al., 2023).

Alternatively, this discrepancy between the genotype and phenotype may result from amino acid substitutions in F731’s MCR-8 that affects its conformation. Indeed, these mutations occur in essential regions such as the phosphoethanolamine transferase N-terminal domain (amino acids 58 to 210), which includes the active site for enzymatic activity, potentially causing partial or complete inactivation of the MCR-8 enzyme. Similar effects may arise from substitutions in the sulfatase N-terminal domain (amino acids 236 to 529), also involved in enzymatic activity (Gaballa et al., 2023).^11^ In some cases, a single mutation can determine antimicrobial susceptibility, as illustrated by MCR-5, where a substitution of Ser284 by Asp reduces colistin resistance (Suzuki et al., 2014; Joshi and Matange, 2024). Even if the expression of *mcr-8.6* is associated with susceptibility to colistin, in future, possible exposure to colistin selection pressure may not only lead to new adaptive mutations, but also to new genomic rearrangements, which both or individually may restore or enhance resistance functions (Lee et al., 2016).

Within the *Klebsiella* genus, many strains have evolved to become a significant clinical and public health threats worldwide, driven by multidrug resistance, and recently, also hypervirulence (Xu et al., 2024). Virulence-associated genes were identified in F731, particularly those linked to biofilm formation, host-cell adhesion, and nutrient uptake via siderophores, all of which have been associated with severe clinical infections (Xu et al., 2024). Additionally, mutations affecting quinolone resistance (*gyrA* and *parC*), porin function (*omp*K36), and efflux pump regulation (e.g., *acrAB*, *oqxAB*, *ramA* and *rarA*), some of these found in F731 (such as *acrA*, *acrB*, *ompK*, *oqxA*, *oqxB*, *ramA*), are also frequently reported among the emergence of multidrug-hypervirulent *Klebsiella* spp (Dai and Hu, 2022; Dong et al., 2022). However, it is important to note that the mere presence of virulence genes does not guarantee that these will be expressed. They are only activated under certain circumstances, such as when the host is immunocompromised, allowing the bacteria to enter a pathogenic state, which is concerning, as it remains a potential threat for these patients (Xu et al., 2024).

**FIGURE 4:**
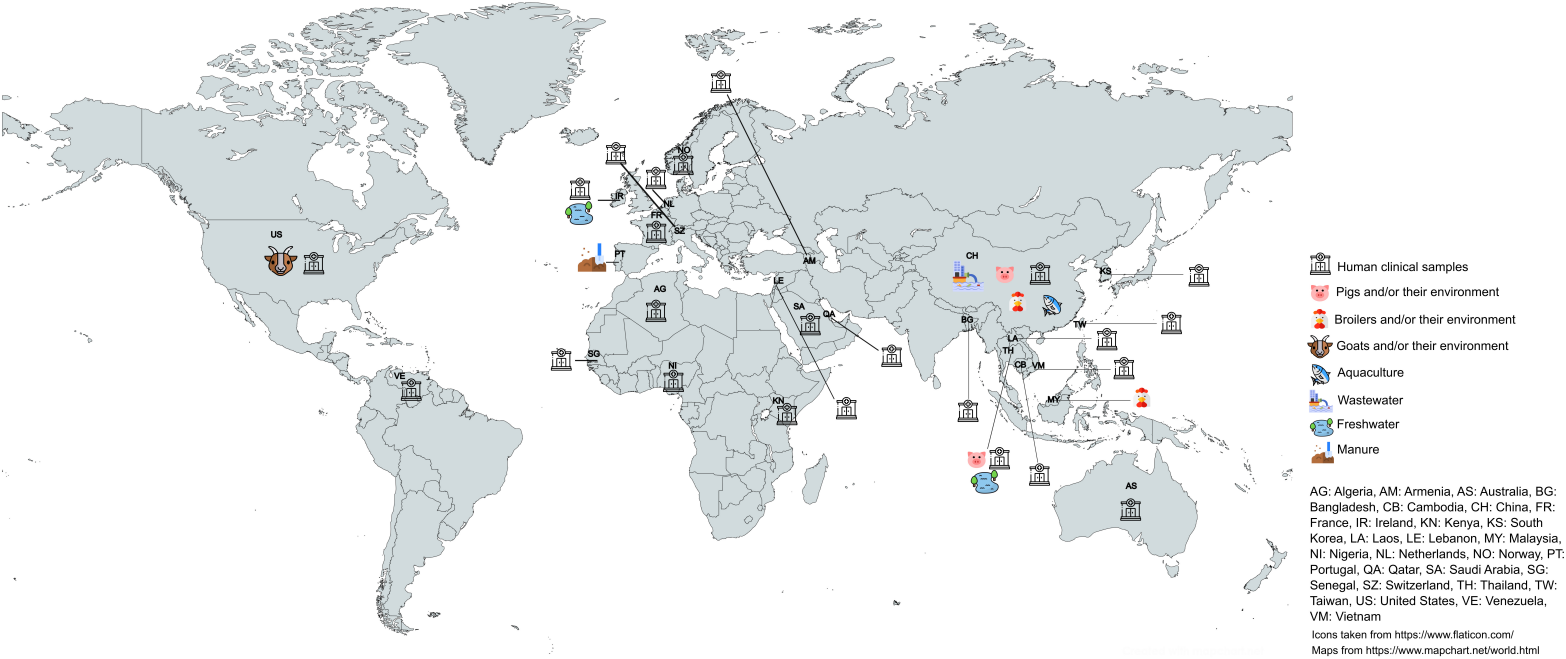
Global distribution of *mcr-8* across sources. Data correspond to reports published between 2018 and 2025.

Colistin has been extensively used in both human and veterinary medicine worldwide, especially in pig farming, where it is applied for the prevention, treatment of diseases, and as a growth promotor (Kempf et al., 2016; Rhouma et al., 2016). Since 1st January 2006, however, the use of antibiotics as growth promotors were banned for the as growth promoters has been banned in the European Union [Regulation (EC) 1831/2003]. Outside of Europe, colistin continued to be used as a growth promotor until later, such as China who only prohibited in 2017. In the European Union, colistin use was restricted to veterinary therapeutic purposes (Walsh and Wu, 2016). In 2016, the European Medicines Agency (EMA), advised to minimize colistin for animals and restrict its use to last-resort situations (European Medicines Agency, 2016), and since then, several countries, such as Denmark, Spain, the UK, Italy, and Portugal, introduced voluntary bans or significant restrictions during the following years (DGS et al., 2019; Ahmed et al., 2025). Since January 2022, European Union regulations on veterinary medicines [Regulation (EU) 2019/6] and medicated feed [Regulation (EU) 2019/4] have banned for routine use of antimicrobials in farming, including preventive group treatments (More, 2020).^12^,^13^ Despite these restrictions, colistin resistance genes, especially plasmid-mediated *mcr* variants, continue to be reported worldwide, posing a challenge to clinical use of this antimicrobial as a last resort treatment. In Portugal, *mcr-1* is the most prevalent variant of plasmid-mediated colistin resistance genes, being frequently detected in human, animal and environmental samples, reflecting the selective pressure induced by prior colistin use across several reservoirs (Mendes et al., 2018; Manageiro et al., 2020; Amaro et al., 2023). Other variants such as *mcr-4* (Amaro et al., 2023) and *mcr-9* (Manageiro et al., 2022) have also been detected. The *mcr-8* gene has already been detected in Europe, specifically in Ireland, France and the Netherlands (Cahill et al., 2023; Zhang et al., 2025). However, our findings add new evidence to the growing list of *mcr* subvariants with the first report of *mcr-8.6* in Portugal (Figure 4).

Chromosomal encoded resistance genes typically imply a reduced immediate risk of transmission compared to those carried on plasmids. Nonetheless, in F731, the *mcr-8.6* subvariant is chromosomally located within a genomic island, which may facilitate mobilization and highlights the potential risk for HGT dissemination (Riquelme et al., 2023). Genomic islands play a significant role in bacterial adaptation and evolution, and typically consist of two key regions: (i) a “multidrug resistance region,” enriched with integrons, insertion sequences, and transposons that facilitate ARG acquisition and incorporation; and (ii) a “core region,” containing genes essential for the island’s stability and maintenance. Thus, genomic islands not only carry ARGs but also the necessary machinery for their mobilization (Morita et al., 2020; Ilyas et al., 2024). Indeed, the genomic island carrying *mcr-8.6* in F731 harbors multiple insertion sequences, such as IS*110*, IS*66*, IS*630* and IS*3*, as well as an integrase gene, suggesting previous mobility events. In fact, e.g., the IS*110* family transposases has been described to co-occur frequently with Tn*3* in bacterial resistance islands and are found integrated in plasmids, where its specific mode of action may facilitate excision, formation of a circular double-stranded DNA, and integration into the target DNA sequence (Durrant et al., 2024; Hiraizumi et al., 2024; Wang and Dagan, 2024).

Furthermore, the high degree of sequence identity of the regions flanking the new *mcr-8.6* gene among both plasmid and chromosomal sequences highlights the potential mobility of *mcr* genes. However, the total sequence of the genetic island has not been identified previously in other strains. Notably, plasmids can integrate into the chromosome via site-specific recombination events involving insertion sequences, e.g., IS*3*, present in the F731 genomic island (Mark Osborn and Böltner, 2002). The presence of a tyrosine-type recombinase/integrase further supports that the genomic island may have originated from another MGE, as these enzymes enable excision from the chromosome in a *recA*-independent manner, a mechanism commonly associated with plasmids, phages and integrative elements (Mark Osborn and Böltner, 2002). However, classic plasmid housekeeping genes (e.g., *rep*, *par*, *tra*, *mob*) were not detected in F731, emphasizing that the presence of integrases and insertion sequences alone is insufficient to confirm a plasmid origin (Mark Osborn and Böltner, 2002).

*K. michiganensis* strain F731 was detected during an annual longitudinal study conducted at an Open Air Laboratory, that covered a crop growing cycle, involving interconnected environmental compartments along the following pathway: pig farm → manure → soil → crop/food/feed → ground/surface water → pig farm. F731 strain was isolated from manure, which highlights the critical role of manure as a potential high-risk reservoir-of resistance determinants in agricultural soils and along the food/feed chain. Importantly, the detection of *mcr-8.6* in this matrix raises concerns about the potential spread of this ARG beyond the farm environment. The use of manure as fertilizer may facilitate the dissemination to bacteria in soil, water, crops and animal microbiota, and thereby, increasing the risk of transmission to human via different pathways (e.g., food and water consumption, occupational exposure, animal contact). Such environmental reservoirs can serve as a bridge between agricultural and clinical settings, contributing to the spread of resistance determinants into healthcare (Lima et al., 2020; Marutescu et al., 2022).

## 5 Conclusion

In this study, we characterized the antimicrobial resistance profile of a *K. michiganensis* strain recovered from manure in Portugal. The detection of a novel chromosomal *mcr-8* subvariant, *mcr-8.6*, marked the first report of this gene in Portugal, and in a *K. michiganensis* worldwide. Although F731 remained susceptible to colistin, the occurrence of new mutations could potentially lead to the development of a resistance phenotype in the future. The *mcr-8.6* gene was located within a putative genomic island adjacent to other MGEs (e.g., IS*110* and IS*3*), indicating past HGT events and highlighting the potential of future mobilization of this ARG across different bacteria species. In addition, the high similarity of the regions flanking *mcr-8.6* with both chromosomal and plasmid sequences from other bacterial strains emphasize the genetic plasticity of MGE associated with *mcr* genes. Collectively, these results highlight the diversity of *mcr* gene variants and subvariants and their association with MGEs, as observed by the genetic context of *mcr-8.6*.

Overall, these findings reinforce the need for continued monitoring and surveillance in environmental and animal compartments of antimicrobial resistance. Furthermore, it also calls for tracking the spread of ARGs over time, through their environmental risk assessment, to identify emerging variants and to map transmission pathways between environmental, animal and clinical reservoirs. Such efforts are essential to preserve last resort antimicrobials, including colistin.

## Data Availability

The datasets presented in this study can be found in online repositories. The names of the repository/repositories and accession number(s) can be found in this article/Supplementary material.

## Footnotes

1
https://doi.org/10.5281/zenodo.5377559

2
https://doi.org/10.5281/zenodo.5376425

3
https://doi.org/10.5281/zenodo.5381928

4
http://www.eucast.org/clinical_breakpoints/

5
https://github.com/B-UMMI/INNUca

6
http://www.bioinformatics.babraham.ac.uk/projects/fastqc/

7 https://www.ncbi.nlm.nih.gov/datasets/genome/?taxon=570, consulted on January 15, 2025

8
https://github.com/tseemann/abricate

9
https://github.com/moshi4/pyGenomeViz

10 https://www.ncbi.nlm.nih.gov/pathogens/refgene/#mcr, consulted on January 22, 2025

11 https://www.uniprot.org/, consulted on July 9, 2025

12
https://eur-lex.europa.eu/eli/reg/2019/6/oj/eng

13
https://www.eubusiness.com/health/vmp/
